# Advancement in biomedical implant materials—a mini review

**DOI:** 10.3389/fbioe.2024.1400918

**Published:** 2024-07-03

**Authors:** Ashish Daniel S., Suya Prem Anand P., Jesuarockiam Naveen, Tabrej Khan, Shabir Hussain Khahro

**Affiliations:** ^1^ School of Mechanical Engineering, Vellore Institute of Technology, Vellore, India; ^2^ Department of Engineering Management, Faculty of Engineering, Prince Sultan University, Riyadh, Saudi Arabia

**Keywords:** biomaterials, implants, bio-compatible, metal alloys, ceramics, polymers

## Abstract

Metal alloys like stainless steel, titanium, and cobalt-chromium alloys are preferable for bio-implants due to their exceptional strength, tribological properties, and biocompatibility. However, long-term implantation of metal alloys can lead to inflammation, swelling, and itching because of ion leaching. To address this issue, polymers are increasingly being utilized in orthopedic applications, replacing metallic components such as bone fixation plates, screws, and scaffolds, as well as minimizing metal-on-metal contact in total hip and knee joint replacements. Ceramics, known for their hardness, thermal barrier, wear, and corrosion resistance, find extensive application in electrochemical, fuel, and biomedical industries. This review delves into a variety of biocompatible materials engineered to seamlessly integrate with the body, reducing adverse reactions like inflammation, toxicity, or immune responses. Additionally, this review examines the potential of various biomaterials including metals, polymers, and ceramics for implant applications. While metallic biomaterials remain indispensable, polymers and ceramics show promise as alternative options. However, surface-modified metallic materials offer a hybrid effect, combining the strengths of different constituents. The future of biomedical implant materials lies in advanced fabrication techniques and personalized designs, facilitating tailored solutions for complex medical needs.

## 1 Introduction

The implant materials are subjected to *in vivo* and physiological conditions which leads to interaction with the cells, tissue growth, and body solutions (Jin and Chu, 2019). Therefore, the implant materials require bioactive properties that influence the growth of tissue around the implant materials. This bioactive property was influenced by surface engineering and surface modification techniques on the bioinert materials. In addition, the hardness, wear, and corrosion resistance of the implant materials were improved when compared to the bioinert material (Unune et al., 2022). Selecting the implant materials according to the application demands leads to reducing the risk to the patient and avoiding secondary surgeries. The implant materials are differentiated into two main applications, namely, temporary and permanent implants. In temporary implant applications, the devices such as bone fixation plates, screws, nails, and wire are used for an implant. Load-bearing applications such as hip joint, knee joint, ankle, and prosthesis come under permanent application (Jin and Chu, 2019). Biodegradable materials are the best choice for temporary implants because they avoid secondary surgeries to remove the implant materials after the healing period (Peng et al., 2019). On the other hand, permanent application requires non-degradable materials with high mechanical, tribological resistance, and biocompatibility properties to avoid premature failure, and inflammation causes for the patients. The biomaterials such as metals alloys, ceramics, and polymers are used for these temporary and permanent implants based on their application needs. Each material has its unique mechanical, tribological, and biocompatibility properties, all these properties are attained in metallic alloys, ceramic, and polymer-based materials, and these materials can withstand biological function and physiological conditions for orthopaedic application. For instance, metal alloys are most frequently used in load-bearing applications because of their strength (Davidson and Mishra, 1992), whereas polymers are used as a barrier in the metal-on-metal contact area to reduce friction and wear debris (Hussain et al., 2020). In addition, ceramics are used for good corrosion and wear resistance, and bioactive ceramics are employed for enhancing the material performance by coating (Zang and Xun, 2021). This minireview critically analyzed the potential of biocompatible materials, metallic, ceramic and polymeric materials for implant applications.

## 2 Bio-compatible property

**FIGURE 1:**
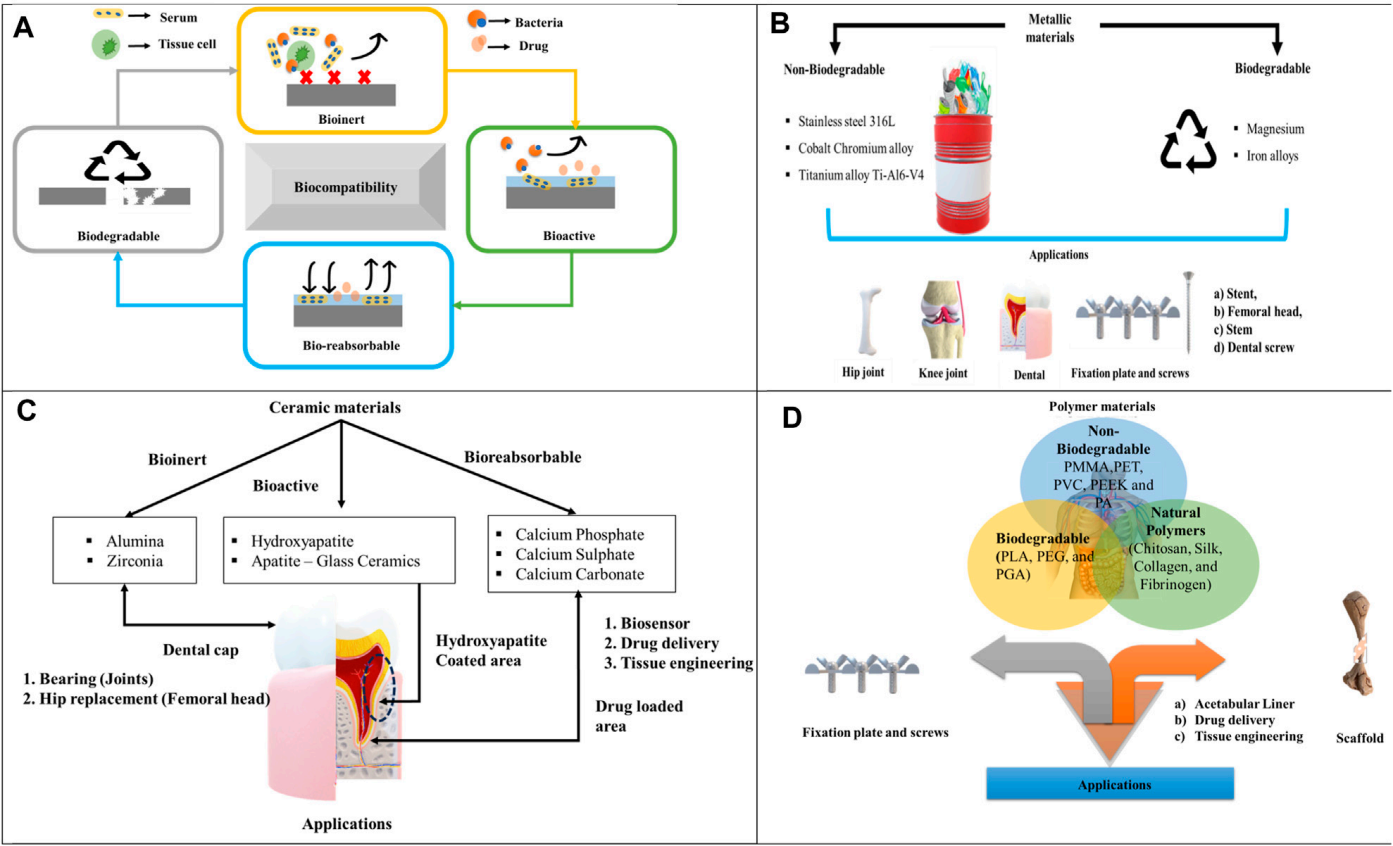
**(A)** Diagrammatic representation of biocompatible behavior, **(B)** Metallic materials, **(C)** Ceramic materials, **(D)** Polymer materials.

Nowadays, the need for bio-implant increases for different patients, which includes design, innovative structure, and manufacturing of a bio-implant as a challenging task to develop its complex structure. Materials consist of different properties such as bioinert, biodegradable, bioactive, and bioresorbable to achieve the desired function of the implant in a body. The implant materials should be biocompatible to perform a certain function in a body. The diagrammatic representation of biocompatible material is shown in Figure 1A. The term bio manufacturing refers to the combination of life science and basic engineering to form a product that is biocompatible to enhance the quality of life science. However, the concept is to make composite material, which consists of an integration of synthetic (artificial) and biological materials like protein cells (Bartolo et al., 2012).

### 2.1 Bioinert

The bioinert material can lower the immune reaction and foreign body reaction in the implanted areas. Due to the foreign body interaction, the immune reaction occurs on the implanted materials by forming the fibrous capsule favors, but the implant material has a poor tissue interface (Castner and Ratner, 2002). Hence, the implant fails and undergoes multiple surgeries. Here, researchers have used two approaches such as biological fixation and bioactive fixation to overcome implant failures (Cao and Hench, 1996). In biological fixation, the implant material surface is modified with a porous structure and rough surface to improve tissue ingrowth and angiogenesis. On the other hand, bioactive fixation improves the strong mechanical bond in the bone-implanted area (Salinas et al., 2013). Metallic materials such as titanium, stainless steel, cobalt chromium are employed as bioimplant in specific application like stem, stent, fixation plate and screws (Pandey et al., 2020). Also ceramics like zirconia and alumina are used as load bearing material for hip joint femoral head, joints, and dental cap applications.

### 2.2 Bioactive

Generally, bioactive materials are manufactured to enhance the biological response and cell behavior (Navarro et al., 2008). In the biological environment, the bioactive materials help to avoid fibrous layer formation and prosthesis failure, it also provides consistency to the bone growth and mineralization between the natural bone and artificial bone (implant material). The major factors concerned in implantation are the mechanical properties and the adhesive bond between the tissue and implant material because it helps to enhance the life of the implant material and avoid multiple surgeries (Hench, 1998). Significantly, the pores design factor also plays a major role in cell growth and colonization, but the void pores structure causes impaired vascularization due to endothelial cells (Salem et al., 2002). In addition, large pores affect the integrity of the material (Karande et al., 2004). On the other hand, pores less than 100 nm affect the material factor and nutrients, which causes implant failure and push to multiple surgeries (Zimmermann et al., 2004). Hydroxyapatite, and glass ceramics belongs to bioactive group, which are used as coating on the surface of metallic material to improve the surface property of the implants (Kumar and Singh, 2024; Priyanka et al., 2024).

### 2.3 Bioresorbable

The material that has the potential to degrade in the physiological environment without causing any toxic effect on the patient is called bioresorbable or bioabsorbable material. Due to the biocompatibility of the bioresorbable material, advanced technologies contributed to the development of the material by using different syntheses, implant designs, and innovative surgical equipment (Freed et al., 1994). Calcium phosphate, sulphate and carbonate are considered as bioresorbable, where these materials are used for the drug delivery application.

### 2.4 Biodegradable

Biodegradability is the property of a material to degrade over a period when present in the human body. The biodegradable materials are converted into other elements like CO_2_, water, and iron by the action of microorganisms. Different biodegradable materials are considered for the temporary bone fixation process. These materials consist of magnesium, Fe, and zinc-based alloys in metallic implants. Polyethylene glycol (PEG) and Polyglycolide (PGA) are included as biodegradable materials in the polymer category.

## 3 Metallic materials

Metallic biomaterials such as Stainless Steel (SS 316L), Titanium alloys (Ti-6Al-4V), and Cobalt Chromium (Co–Cr) are predominantly employed to replace defective hard tissue. These materials have been extensively studied due to their suitable bulk properties (Al-Amin et al., 2020). The classification of different metallic materials is shown in Figure 1B.

### 3.1 Non-biodegradable metals

#### 3.1.1 Titanium alloys

Titanium alloys possess unique properties including exceptional mechanical, corrosion resistance, and biocompatibility with low-density factors. Combining titanium with former metals improves the biocompatible of titanium alloys that are extensively utilized in the manufacturing of bioimplants. Out of these materials, the titanium alloys have different crystal structures like α+β and low young modulus β phase match near to natural bone properties. This material with a specific crystal structure is recognized as a suitable replacement for hard tissue without disclosure of toxic elements (Niinomi, 2002). The elements present in titanium alloys such as (Ta)Tantalum, (Nb) Niobium, and (Zr) Zirconium are considered as harmless which are essential for the strength of bioimplants. It has been proved that a low modulus of titanium alloy can be used to replace natural bone fracture. Ti-6Al-4V alloy is the recommended material for different application in the biomedical field and includes 45% of implant production. As, Ti alloys consist of elastic modulus above that of a natural bone (55–110 GPa) (Majumder et al., 2020) the stress shielding effect can be reduced compared to other materials.

#### 3.1.2 Stainless steel alloys

Stainless steel (SS 316L) is a highly recommended metal alloy for biomedical applications because of their mechanical properties and low manufacturing cost (Bekmurzayeva et al., 2018). SS 316L contains 10.5% of chromium elements which helps to alter the surface by forming the metal chromium oxide layer and improving the corrosion resistance. Moreover, SS 316L contains different elements such as iron, nickel, and carbon. The presence of 0.03% carbon in the steel alloy maximizes the yield strength and corrosion resistance for orthopedic applications (Lodhi et al., 2019). Duplex steel is also preferred as an implant by altering the surface of the material using specific surface modification technique. This enhances the longevity of the implant for biomedical application (Pandey et al., 2020; Mahajan et al., 2023a).

#### 3.1.3 Cobalt-chromium alloy

Cobalt-Chromium (Co-Cr) alloys are precisely used in load-bearing applications such as hip and knee joints due to their hardness, and tribological properties (Mahajan et al., 2023a). In addition, clinical studies proved that the Co-Cr alloys are good in biocompatibility. In Co-Cr alloys, chromium contains 28 wt%, 6 wt% Mo, and balance wt% of Co and it interacts with cells, and protein solutions in the human body (Bandyopadhyay et al., 2019b; Bandyopadhyay et al., 2019b). The presence of 28 wt% Cr leads to the formation of Cr_2_O_3_ oxide layer on the material surface which shields the surface from corrosive environment, also it enhances biocompatibility and improves osseointegration (Alvarez-Vera et al., 2021).

### 3.2 Biodegradable metals

#### 3.2.1 Magnesium alloys

Magnesium-based alloys are recently considered to reduce the difficulties of long-term bioimplants in the human body due to their biodegradable property. After the material degradation, the destroyed elements are further processed to satisfy the basic demands of metabolic pathways. Earlier titanium β-type alloy is used for long-term application due to its strength, low Young’s modulus, and biocompatible. Among different metallic alloys, magnesium is currently focused on replacement joints, knee joints, bone plates, and shoulders (Ibrahim et al., 2017). Moreover, this material is also applicable for cardiovascular stents, trauma, spinal discs, and fixation plates (Rajan Soundararajan et al., 2018). The implementation of low-cost β-type titanium alloy is increased for implant application because of having high biocompatibility by alloying with other elements. This type of material is formed by a cold working process (Yumak and Aslantaş, 2020).

Currently, in metallic materials, magnesium plays an important role in the biomedical field due to its biodegradable properties. This tries to avoid secondary surgery from the clinical perspective. Although the usage of magnesium increases in implant applications, there is an issue with a faster corrosion rate within the human body over some time (Chen et al., 2019). To overcome the limitations of commercially available magnesium, it is recommended to use magnesium alloys such as magnesium zinc, magnesium copper, and magnesium calcium (Uva Narayanan et al., 2023).

### 3.3 Ceramic materials

Bio-ceramic material is increasingly used in the medical field due to its excellent properties like biocompatible, mechanical strength, aesthetic look, chemical stability, and porous structure (Petit et al., 2018). Here, the challenging part is the interaction of ceramic material with bone tissue, where the ceramic material is biologically stable or reabsorbed over a period. The idea of using bio-ceramic scaffolds, coating ceramic materials, and composite materials changed over a period without altering the biological properties (Pradeep et al., 2022). Nowadays, ceramic materials such as alumina, zirconia, and zirconia-toughened alumina are used to replace fractured bone in orthopedic applications. In addition, additive manufacturing technology is used to make these ceramic materials efficient compared to the conventional subtractive method (Liu et al., 2022). Different materials for ceramic materials are shown in Figure 1C.

#### 3.3.1 Bioinert and bioactive

The ceramics employed in orthopedic applications are classified into two types such as bioinert and bioactive depending upon the behavior of the material. Therefore, the inert type ceramics are fabricated dense for bearing in the joint replacement to improve the strength of the material against wear and corrosion conditions. The total joint hip replacement includes the metal-on-polyethylene combination, where the long-term reliability of joints in the human body depends upon the wear of its metal component (Todros et al., 2021). In the long term duration, the polyethylene induces macrophages that lead to the bone reabsorbing cytokines resulting in the loss of bone stocks. On the other hand, bioactive ceramics are biologically bonded to the bone, where it is used as a coating material to enhance the fixation of the device because of the osteoconductive property (Uva Narayanan et al., 2023; Bera et al., 2024).

However, the combination of alumina on alumina is also proposed owing to exceptional wear and corrosion resistance of the material but resulted in the loosening of the acetabular product for long-term usage. The major problem with ceramic material is fracture that leads to damage of components when consumed for a long period in the human body. Now the risk of brittle fracture is eliminated by implementing new techniques in the fabrication process. Here the microstructure of the ceramic material depends upon the fabrication process, grain size, distribution of the grains, density, and powder quality (Rony et al., 2018).

#### 3.3.2 Bioreabsorbable

The possibility of using ceramics increases in the biomedical field, by improving the surface characteristics of advanced ceramics such as calcium phosphate, silica, and oxide. These ceramics are frequently used in different applications like bio-materials, bio-sensors, implants, drug delivery, and tissue engineering. The wide challenges experienced by the material surface are addressed to overcome the material defects (Treccani et al., 2013). The most essential criterion while developing a biomaterial is to understand the characteristics of the materials towards different bio environments. It also provides detailed information about the behavior of the material such as bio-inert, bio-active, bio-absorbable, biocompatible, and sterilization (Paul, 2019). The nano-structured ceramic materials, coatings, and cement are applied to orthopedic, medical, and dental applications to improve biomedical functions. Recently, ceramic nano-materials such as tri-calcium phosphate, hydroxyl apatite, calcium phosphate, calcium sulfate, bioactive glasses, zirconia ceramics, and alumina are considered for these applications (Balasubramanian et al., 2017).

### 3.4 Polymer materials

Earlier, there are many thermoplastic polymers such as polylactic acid, polymethyl methacrylate, polyvinyl chloride, Polyether ether ketone (PEEK), Polyethylene terephthalate (PET), and poly ethylene existed in the biomedical field owing to their outstanding properties (Ranjan, 2023). Different materials for polymers are shown in Figure 1D.

#### 3.4.1 Non-biodegradable

Among different polymers, polyethylene is predominantly consumed by bioimplants because of its tribological properties. However, it has been stated that the wear particles of polyethylene lead to the failure of artificial joints which causes aseptic loosening. Therefore, research area related to the wear of artificial joints develops into an essential field within implant manufacturing. In the future, newly developed materials such as crosslinked with polyethylene, bioceramics, and fiber-reinforced PEEK with enhanced wear resistance and outstanding biocompatibility, are recommended for the fabrication of artificial joints. Lubricant may be considered an important parameter and its purpose is to duplicate the synovial fluids. The chances of high pressure being developed between the joints with a sliding velocity tend to increase the wear of the material. Moreover, the hard particles might scratch the bearing surface, leading to the rise of wear and tear. Artificial joints for total hip and knee are the most popular applications for these polymers (Gang et al., 2018).

#### 3.4.2 Biodegradable (natural polymers)

At present, there is a chance of adding chitosan as filler to the PVC polymers to improve the biocompatible behavior of the matrix for implant and scaffold application (Ranjan, 2023). In the current situation, the usage of hyper-branched polymers is increasing in biomedical applications such as drug delivery, tissue engineering, and detecting diseases. This is due to the bio-compatible and degradable properties of the polymers that match the requirements of biomedicine. When compared to nano-sized polymers, these hyperbranched polymers are easy to fabricate (Saadati et al., 2021).

The improvement of polymer composites is discussed which is used in biomedical applications in terms of the fabrication process, structure, and physical properties. The necessities of bioactive polymers are essential for bone regenerative, oxygen-releasing, and conductive materials. Most of the applications associated with the biomedical field are listed as polymer stents sutures, porous membranes, artificial heart valves, and barrier films. On the other hand, non-bioactive polymers are developed to make a scaffold of porous structure which is placed as filler for supporting the fractured bone (Guo et al., 2021). Currently, polymer composites are also used as bio-absorbable films in the medical field such as wound healing, skin treatment, and scaffolding (Prasad, 2021). Different materials and their applications are listed in Table 1.

**TABLE 1:** Different biomaterials and their application.

S. No	Biomaterials	Classifications of biomaterials	Application	Reference
1	Metallic materials	Titanium and its alloy	Dental screw, nail, wire, fixation plate, and stem	Kassapidou et al. (2020)
		Stainless steel and its alloy	Fixation plate, screw, nails, dental, scaffold, stent, femoral head, and stem	Love (2017)
		Cobalt-chromium and its alloys	Dental prosthesis, hip joint, knee joint, stent, ankle prosthesis, and stem	Omar et al. (2017)
		Magnesium and its alloy	Fixation plate, screw, pins, and nails	Kamrani and Fleck (2019)
2	Ceramic materials	Hydroxyapatite	Scaffold, bone cement	Sari et al. (2021)
		Zirconia	Femoral head, scaffold, dental, and bone cement	Zhu et al. (2019)
		Alumina	Femoral head, and dental application	Kamitakahara et al. (2008)
		Calcium phosphate, silica, and oxide	Bio-sensors, implants, and drug delivery	Treccani et al. (2013)
		Tri-calcium phosphate, calcium phosphate, calcium sulfate, bioactive glasses	Coated for orthopedic, dental and medical application	Balasubramanian et al. (2017)
3	Polymer materials	Polylactic acid	Fixation plate, screw, pins, scaffolds, and nails	Wang et al. (2021)
		Ultra-high molecular weight polyethylene (UHMWPE)	Acetabular liner and scaffold	Shahemi et al. (2018)
		Polyetheretherketone (PEEK)	Acetabular liner and scaffold	Heijnens et al. (2021)
		Polymethyl Methacrylate (PMMA)	Bone cement, bone substitute and dental application	Ranjan (2023)
		Hyperbranched polymer	Drug delivery, tissue engineering	Kassapidou et al. (2020)
		Natural polymers (Chitosan, Silk, Collagen, and Fibrinogen)	Scaffold, Implant applications	Ranjan (2023)

## 4 Conclusion

Challenges associated with biocompatible materials include achieving optimal biocompatibility without compromising mechanical strength, durability, or functionality. Additionally, ensuring long-term stability within the biological environment, minimizing immune responses, and addressing issues related to degradation and biocompatibility over time pose significant hurdles. Generally, the metallic alloys are frequently used in the structural application of biomedical such as knee, hip, and dental implants, where high mechanical strength is needed to withstand the loading conditions. Challenges associated with metallic implant materials include corrosion, metal ion release, and potential allergic reactions in patients. Recently, surface coating of metallic alloys with calcium phosphate, hydroxyapatite, and oxide layer has played a major role in overcoming the leaching problem. Another major issue with metal alloy is the stress shielding effect which is due to the increase in the Young’s modulus compared to the natural bone. The problem associated with magnesium alloys are faster degradation rate, where special surface treatment is required to suppress this issue. Ceramic is especially used for specific applications like acetabular cups, dental crowns, scaffolds, and implants. Ceramic implant materials encounter challenges related to their brittleness and susceptibility to fracture, particularly in high-stress environments. On the other hand, polymers are used for bone regenerative, scaffolds, polymer stents, wound healing, and porous membranes in biomedical applications. Polymer implant materials face challenges related to their mechanical properties, such as strength and stiffness, which may not always meet the requirements for load-bearing applications. Moreover, ensuring long-term stability and preventing degradation or wear over time are significant concerns. Finally, the development of cost-effective manufacturing processes and scaling up production while maintaining quality standards remains a challenge in making these materials widely accessible for medical and biological applications.

## 5 Future scope

The future of implant materials lies in the development of advanced biomimetic designs and innovative fabrication techniques, enabling enhanced integration with the body and prolonged durability. Integration of smart materials and nanotechnology holds promise for personalized, regenerative implants capable of real-time monitoring and therapeutic interventions.
